# Supplementary material for: Women’s preferences for caesarean or vaginal birth with a perspective of future fertility: A discrete choice experiment
Source: PLoS One. 2024 Nov 7;19(11):e0310560. doi: 10.1371/journal.pone.0310560 (PMC11542828; doi:10.1371/journal.pone.0310560)

**S1 File. DCE questionnaire**

**Page 1: Introduction and consent**


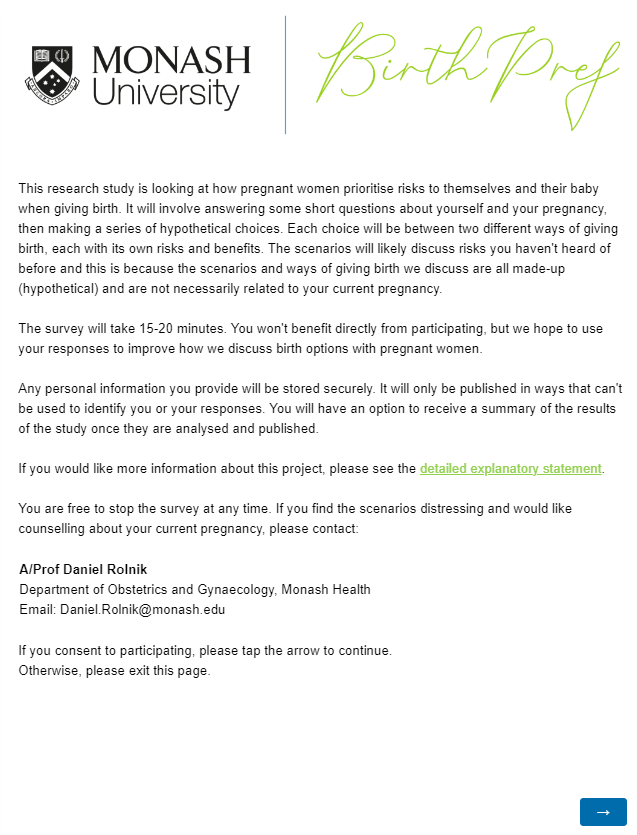


**Page 2: Demographics**


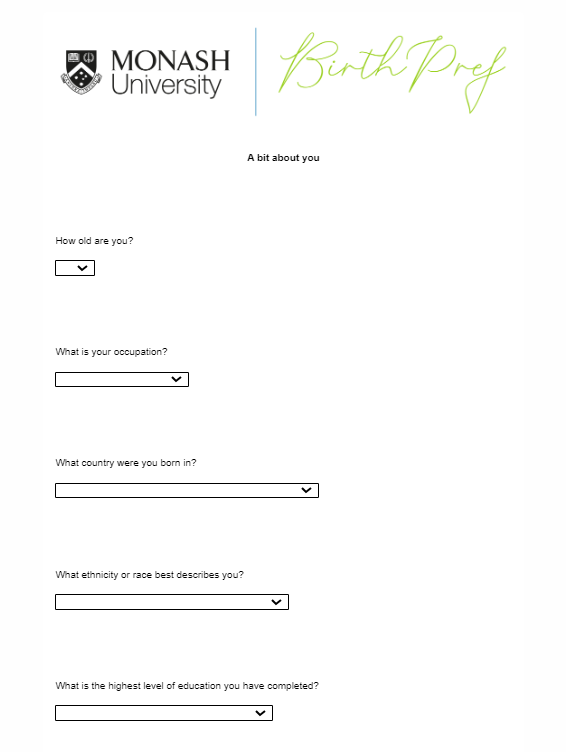


**Page 2 (continued): Demographics**


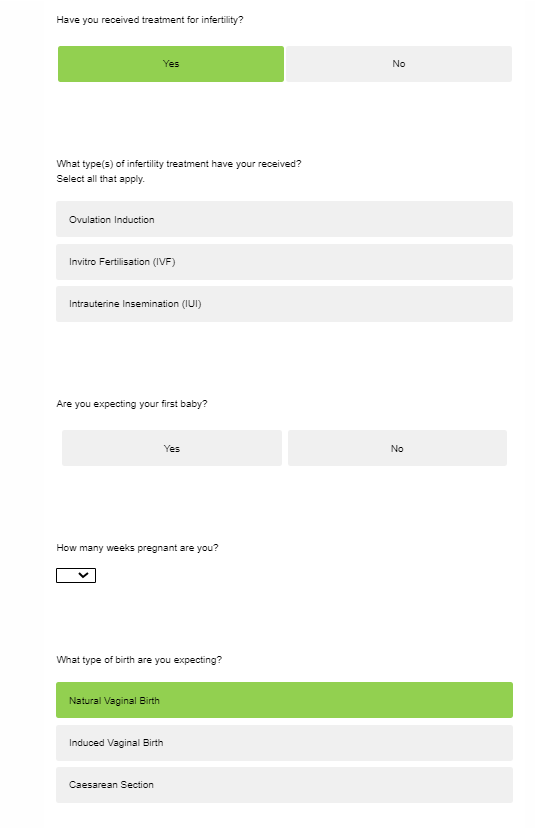


**Page 2 (continued): Demographics**


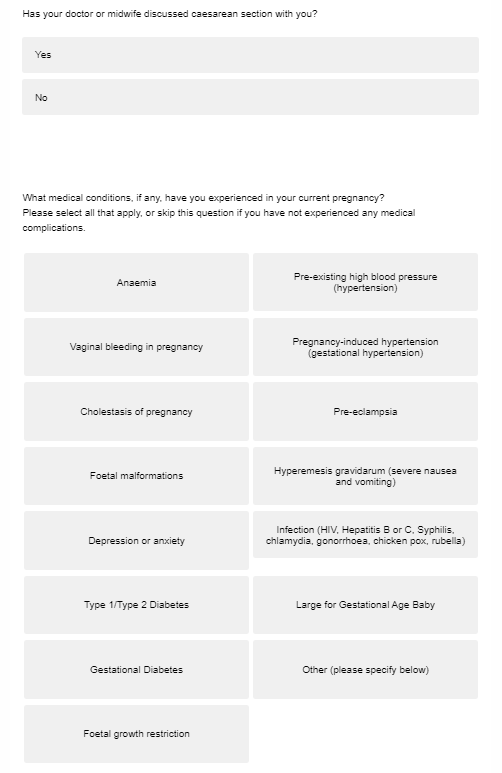


**Page 2 (continued): Demographics**


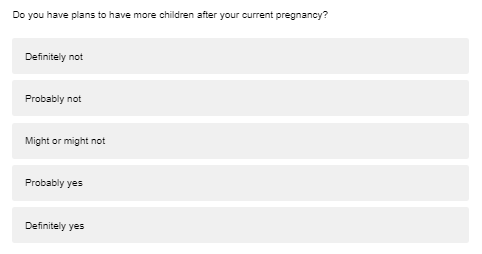


**Page 3: Risk definitions**


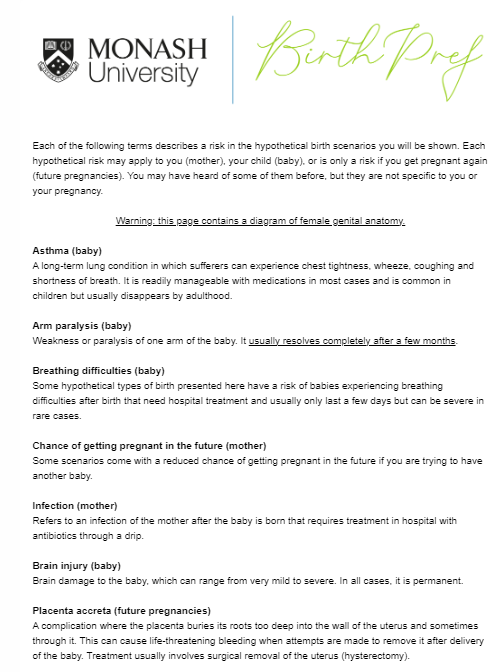


**Page 3 (continued): Risk definitions**


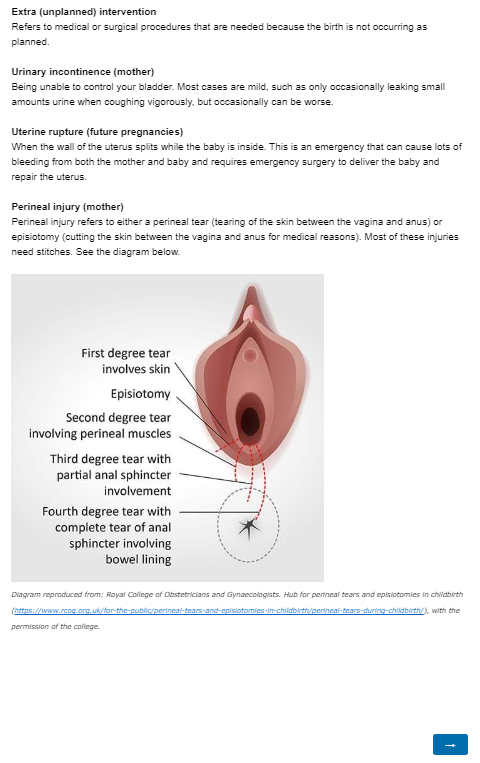


**Page 4: Risk descriptors**


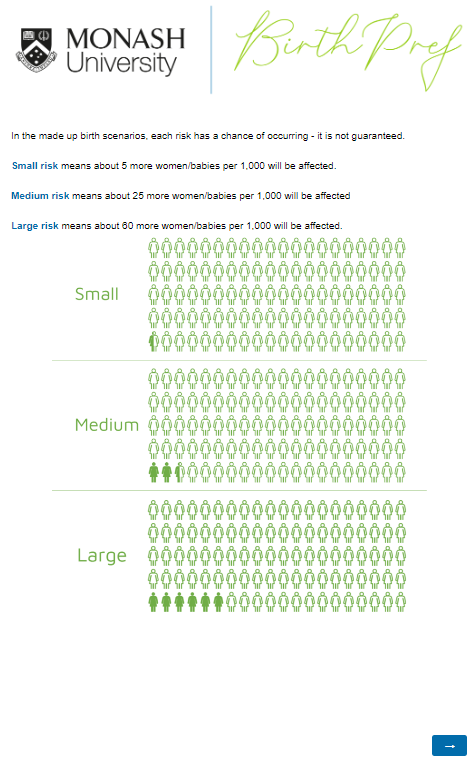


**Page 5: Example choice set**


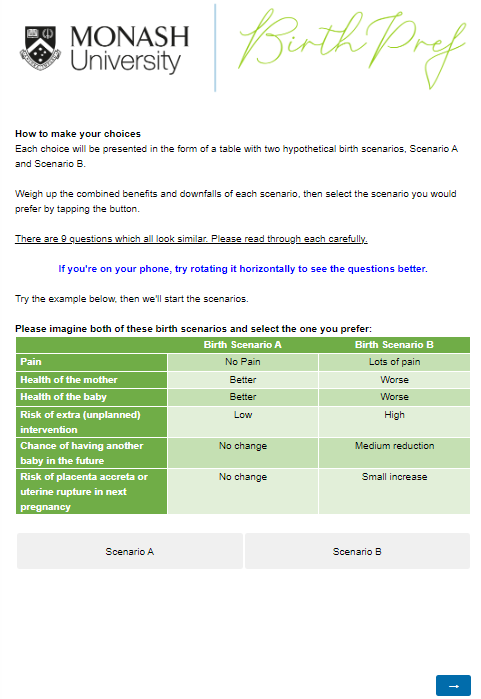


**Page 6: Choice set 1**


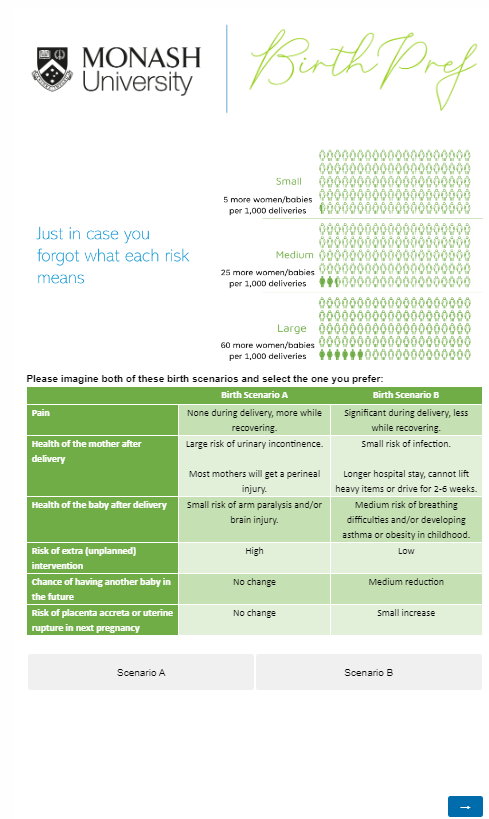


**Page 7: Choice set 2**


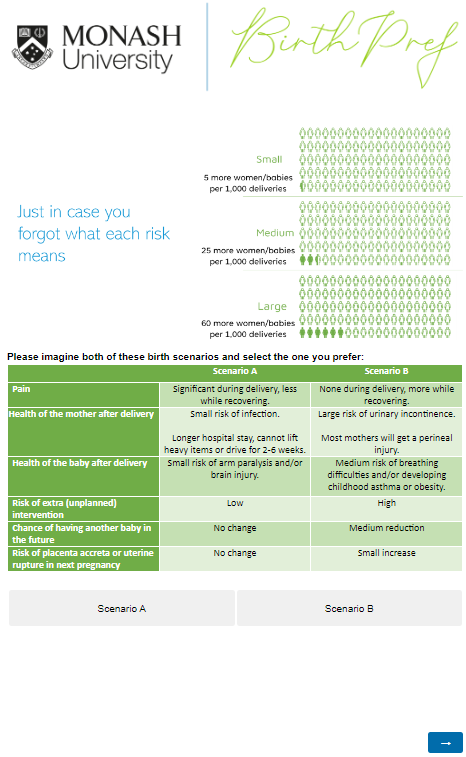


**Page 8: Choice set 3**


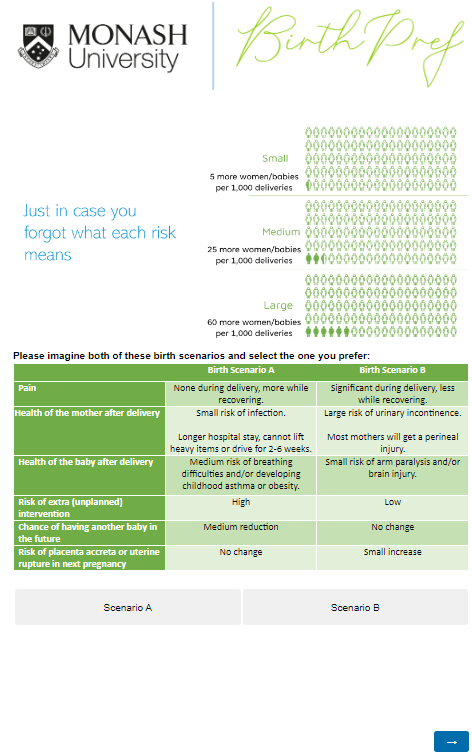


**Page 9: Choice set 4**


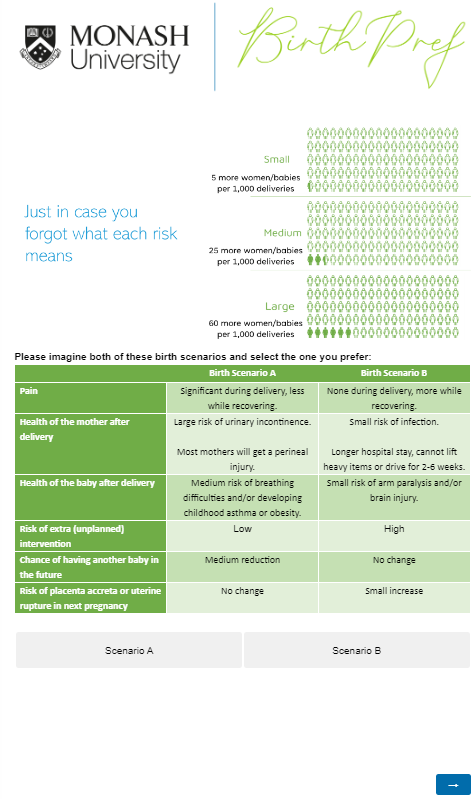


**Page 10: Choice set 5**


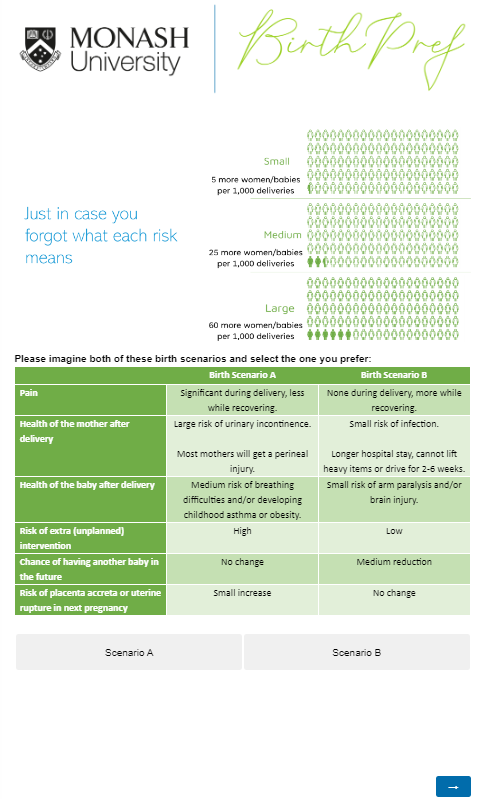
**Page 11: Choice set 6**


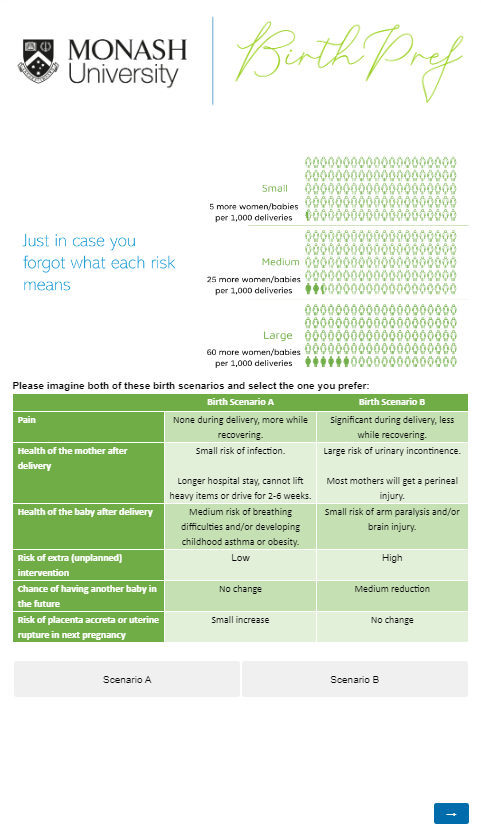


**Page 12: Choice set 7**


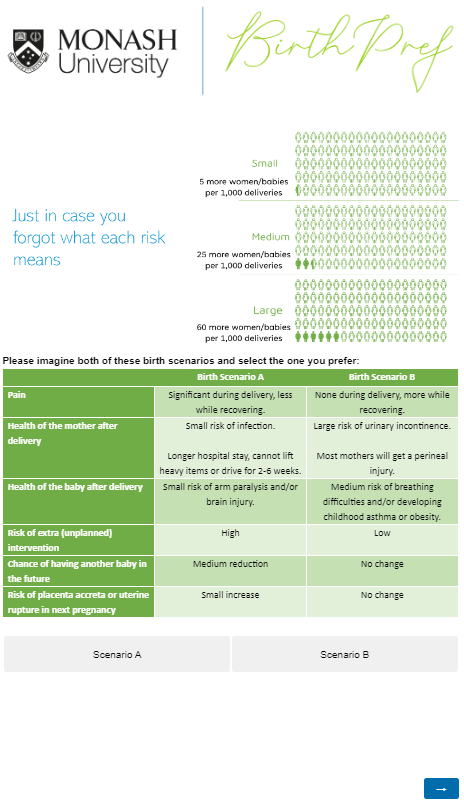


**Page 13: Consistency choice set (identical to choice set 2)**


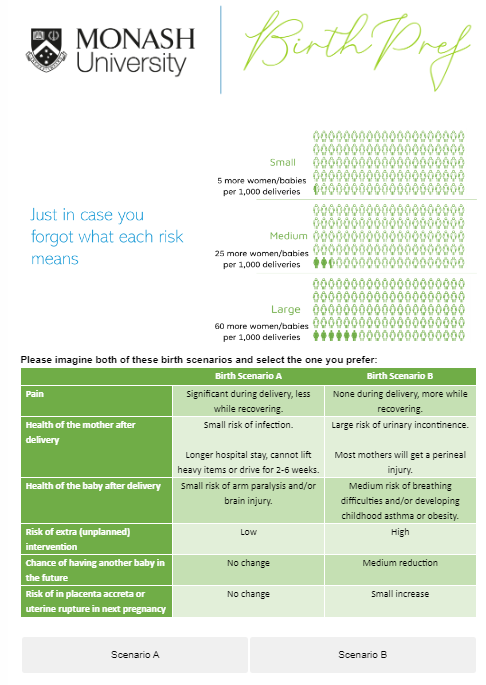


**Page 14: Choice set 8**


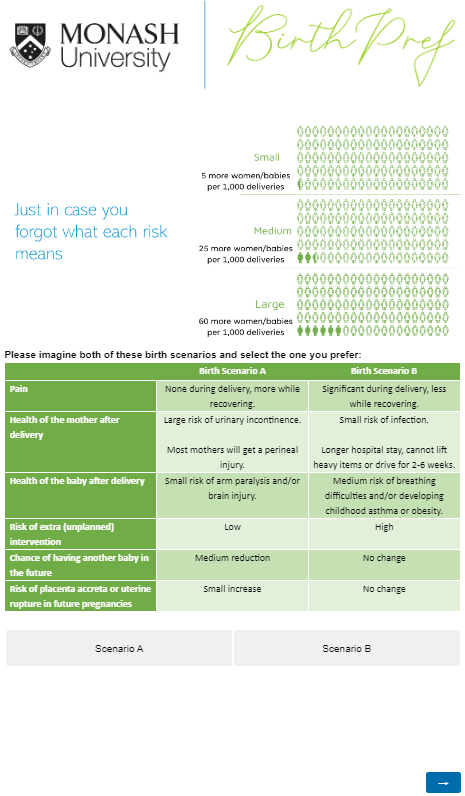


**Page 15: Difficulties and suggestions for improvement**


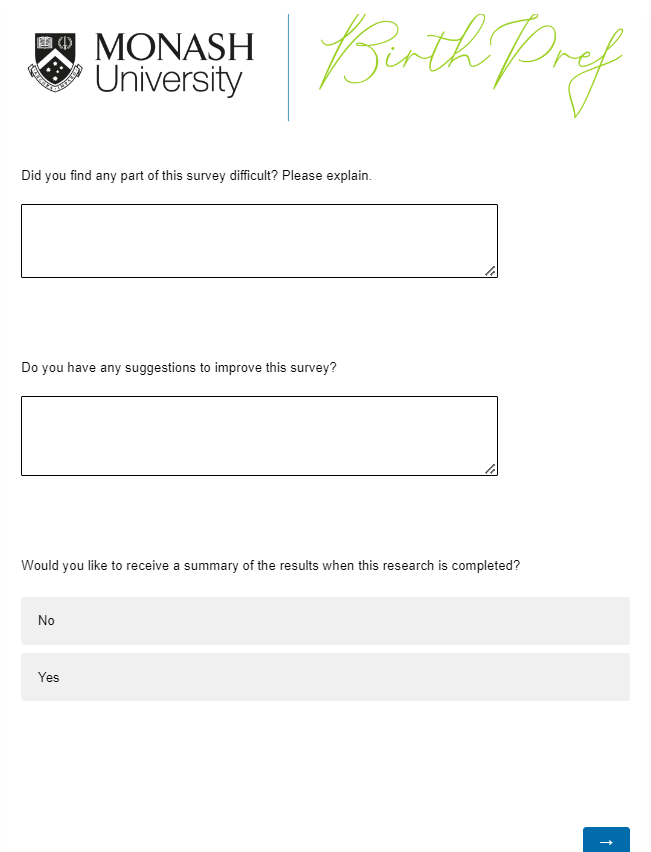


**Page 16: Thank you message**


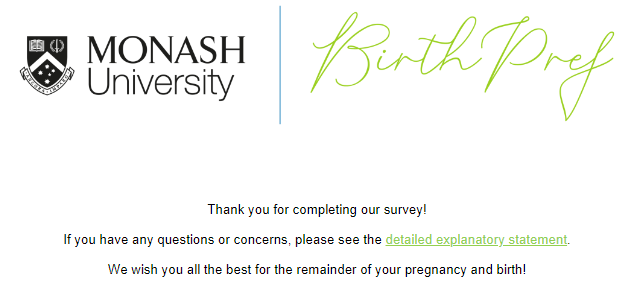

Supplement: S1 File — (DOCX) [file pone.0310560.s001.docx]
