# Supplementary material for: Women’s preferences for caesarean or vaginal birth with a perspective of future fertility: A discrete choice experiment
Source: PLoS One. 2024 Nov 7;19(11):e0310560. doi: 10.1371/journal.pone.0310560 (PMC11542828; doi:10.1371/journal.pone.0310560)
Supplement: S1 Table — (DOCX) [file pone.0310560.s004.docx]

**S3 Table. Content analysis of difficulties reported as free text by respondents.**

| Reported difficulty – n (%) | All participants (N=211) | Interview participants (n=34) | Online survey participants  (n=177) |
| --- | --- | --- | --- |
| No reported difficulties | 139 (65.9%) | 10 (29.4%) | 129 (72.9%) |
| Difficultly choosing between the risks | 30 (14.2%) | 16 (47.1%) | 14 (7.9%) |
| It felt like there were no good options | 8 (3.8%) | 3 (8.8%) | 5 (2.8%) |
| Difficult concentrating as all scenarios were so similar | 23 (10.9%) | 3 (8.8%) | 20 (11.3%) |
| Difficulty understanding medical jargon | 3 (1.4%) | 1 (2.9%) | 2 (1.1%) |
| Preferences changed after answering some scenarios | 1 (0.5%) | 1 (2.9%) | 0 (0.0%) |
| Difficulty understanding risks | 7 (3.3%) | 0 (0%) | 7 (4.0%) |
| Scenarios did not seem realistic | 4 (1.4%) | 0 (0%) | 4 (2.3%) |
| Risks in scenarios were confronting | 3 (1.4%) | 0 (0%) | 3 (1.7%) |
| Did not understand task | 1 (0.5%) | 0 (0.0%) | 1 (0.6%) |
| Unspecified difficulties | 3 (1.4%) | 0 (0.0%) | 3 (1.7%) |

Note that some participants reported multiple difficulties.
