# Supplementary material for: Women’s preferences for caesarean or vaginal birth with a perspective of future fertility: A discrete choice experiment
Source: PLoS One. 2024 Nov 7;19(11):e0310560. doi: 10.1371/journal.pone.0310560 (PMC11542828; doi:10.1371/journal.pone.0310560)
Supplement: S2 Table — (DOCX) [file pone.0310560.s005.docx]

**S4 Table. Content analysis of suggestions for improvement reported as free text by respondents**.

| Suggestion for improvement – n (%) | All participants (N=211) | Interview participants (n=34) | Online survey participants  (n=177) |
| --- | --- | --- | --- |
| No reported suggestions | 184 (87.2%) | 26 (76.5%) | 158 (89.3%) |
| More detailed description of risks | 4 (1.9%) | 1 (2.9%) | 3 (1.7%) |
| Reduce number of risks in each scenario | 3 (1.4%) | 3 (8.8%) | 0 (0.0%) |
| Add function for participants to annotate choice sets | 1 (0.5%) | 1 (2.9%) | 0 (0.0%) |
| Explain the mode of birth associated with each risk | 4 (1.9%) | 0 (0.0%) | 4 (2.3%) |
| Get participants to order how they preference risks rather than using hypothetical scenarios | 2 (0.9%) | 0 (0.0%) | 2 (1.1%) |
| Perform questionnaire face-to-face | 1 (0.5%) | 0 (0.0%) | 1 (0.6%) |
| Make scenarios less similar | 9 (4.3%) | 1 (2.9%) | 8 (4.5%) |
| Make scenarios less confronting | 1 (0.5%) | 0 (0.0%) | 1 (0.6%) |
| Use fewer scenarios | 1 (0.5%) | 0 (0.0%) | 1 (0.6%) |

Note that some participants provided multiple suggestions.
