# Supplementary material for: Women’s preferences for caesarean or vaginal birth with a perspective of future fertility: A discrete choice experiment
Source: PLoS One. 2024 Nov 7;19(11):e0310560. doi: 10.1371/journal.pone.0310560 (PMC11542828; doi:10.1371/journal.pone.0310560)
Supplement: S3 Table — (DOCX) [file pone.0310560.s006.docx]

**S5 Table. Conditional odds ratios for each attribute for all participants, interview participants only, participants who passed the consistency test only and participants ≥30 years of age only.**

| **Attribute** | **Level** | **Associated mode of birth** | **Conditional odds ratios (95% CI)** | | | |
| --- | --- | --- | --- | --- | --- | --- |
|  |  |  | **All participants (n=211)** | **Interview participants (n=34)** | **Participants who passed the consistency test (n=174)** | **Participants ≥30 years of age (n=125)** |
| **Chance of having another baby in the future** | No change | Vaginal | REF | REF | REF | REF |
|  | Medium reduction | Caesarean | 0.75  (0.68 to 0.83, p<0.001) | 0.59 (0.46 to 0.76, p<0.001) | 0.78 (0.70 to 0.87, p<0.001) | 0.77 (0.67 to 0.87, p<0.001) |
| **Risk of placenta accreta or uterine rupture in next pregnancy** | No change | Vaginal | REF | REF | REF | REF |
|  | Small increase | Caesarean | 0.90  (0.82 to 1.00, p=0.045) | 0.87 (0.68 to 1.13, p=0.299) | 0.89  (0.80 to 0.99, p=0.029) | 0.96 (0.84 to 1.09, p=0.530) |
| **Pain** | Significant pain during delivery, less while recovering. | Vaginal | REF | REF | REF | REF |
|  | No pain during delivery, more while recovering. | Caesarean | 0.92  (0.83 to 1.01, p=0.087) | 1.09 (0.84 to 1.41, p=0.536) | 0.91  (0.82 to 1.01, p=0.080) | 0.96 (0.84 to 1.09, p=0.531) |
| **Health of the baby after delivery** | Small risk of arm paralysis and/or brain injury. | Vaginal | REF | REF | REF | REF |
|  | Medium risk of breathing difficulties and/or developing asthma or obesity in childhood. | Caesarean | 1.01  (0.92 to 1.12, p=0.838) | 1.38  (1.05 to 1.77, p=0.019) * | 1.00 (0.90 to 1.12, p=0.965) | 0.98 (0.86 to 1.11, p=0.701) |
| **Health of the mother after delivery** | Large risk of urinary incontinence. Most mothers will get a perineal injury. | Vaginal | REF | REF | REF | REF |
|  | Small risk of infection. Longer hospital stay, cannot lift heavy items or drive for 2-6 weeks. | Caesarean | 1.29 (1.17 to 1.42, p<0.001) | 1.29 (1.00 to 1.66, p=0.054) | 1.25  (1.12 to 1.39, p<0.001) | 1.35 (1.19 to 1.53, p<0.001) |
| **Risk of extra (unplanned) intervention** | High | Vaginal | REF | REF | REF | REF |
|  | Low | Caesarean | 1.37  (1.24 to 1.51, p<0.001) | 1.48  (1.14 to 1.91, p=0.003) | 1.33  (1.19 to 1.48, p<0.001) | 1.42 (1.25 to 1.61, p<0.001) |

Conditional odds ratios represent the conditional odds of choosing the level of each attribute representing caesarean birth over the attribute representing vaginal birth.
