# Supplementary material for: Women’s preferences for caesarean or vaginal birth with a perspective of future fertility: A discrete choice experiment
Source: PLoS One. 2024 Nov 7;19(11):e0310560. doi: 10.1371/journal.pone.0310560 (PMC11542828; doi:10.1371/journal.pone.0310560)
Supplement: Attachment — Submitted filename: BirthPref_Response_To_Reviewers.docx [file pone.0310560.s007.docx]

10 August 2024

Natasha L Pritchard
Academic Editor
PLOS ONE

Dear Dr Pritchard,

We would like to thank you and the Reviewers for your feedback and the opportunity to revise our manuscript. We have endeavoured to address all your concerns and queries in the manuscript and supporting files. Our revisions in response to each of the comments are tracked in the attached documents and summarised below in the order in which the comments were raised.

We hope that our revisions will meet the expectations of the editors and reviewers, and we hope they make the article acceptable for publication.

Thank you for your ongoing consideration.

Sincerely,

James D. Crispin
Department of Obstetrics and Gynaecology
Monash University School of Clinical Sciences
Monash Medical Centre, Melbourne, Australia

**Journal Requirements**

| A. Reviewer comment | Please ensure that your manuscript meets PLOS ONE's style requirements, including those for file naming. |
| --- | --- |
| B. Response | Apologies for these formatting errors and thank you for attaching the style guide to your email. |
| C. Changes made | We have renamed our supplement files, changed the formatting and size of our headings and subheadings, changed our figure and table captions and amended the formatting of our in-text citations accordingly. |
| D. Location of changes | Throughout manuscript. |

| A. Reviewer comment | Thank you for stating the following in the Competing Interests section: "I have read the journal's policy and the authors of this manuscript have the following competing interests: BWM is supported by a NHMRC Investigator grant (GNT1176437). BWM reports consultancy, travel support and research funding from Merck and consultancy for Organon and Norgine. BWM holds stock from ObsEva. The remaining authors, JDC, DLR and MvW declare no competing interests." Please confirm that this does not alter your adherence to all PLOS ONE policies on sharing data and materials, by including the following statement: "This does not alter our adherence to PLOS ONE policies on sharing data and materials.” |
| --- | --- |
| B. Response | I can confirm that this does not alter our adherence to PLOS ONE policies on sharing data and materials. |
| C. Changes made | Addition of the statement *“This does not alter our adherence to PLOS ONE policies on sharing data and materials”* to the cover letter. |
| D. Location of changes | Cover letter. |

| A. Reviewer comment | In the online submission form, you indicated that "Deidentified raw data is available is a single spreadsheet upon request from the author. This was a requirement of our human research ethics committee given the sensitive nature of some of the included questions." All PLOS journals now require all data underlying the findings described in their manuscript to be freely available to other researchers, either 1. In a public repository, 2. Within the manuscript itself, or 3. Uploaded as supplementary information. This policy applies to all data except where public deposition would breach compliance with the protocol approved by your research ethics board. If your data cannot be made publicly available for ethical or legal reasons (e.g., public availability would compromise patient privacy), please explain your reasons on resubmission and your exemption request will be escalated for approval. |
| --- | --- |
| B. Response | We have clarified with our Human Ethics documentation and can provide the deidentified raw data for upload as a supplementary file. |
| C. Changes made | The deidentified raw data has been uploaded as a supplementary file: S6_file. |
| D. Location of changes | S6_File. |

| A. Reviewer comment | Please review your reference list to ensure that it is complete and correct. If you have cited papers that have been retracted, please include the rationale for doing so in the manuscript text, or remove these references and replace them with relevant current references. Any changes to the reference list should be mentioned in the rebuttal letter that accompanies your revised manuscript. If you need to cite a retracted article, indicate the article’s retracted status in the References list and also include a citation and full reference for the retraction notice. |
| --- | --- |
| B. Response | We have confirmed that none of the articles cited in the text have been retracted. We identified some formatting errors in the reference list and citations, which have been corrected in accordance with the PLOS ONE referencing guidelines. |
| C. Changes made | Due to changes made in response to comments below, the reference list has been regenerated and the order of references has been changed. References missing details have been updated; notably, references 31-33 and 35-37. |
| D. Location of changes | Entire reference list has been regenerated.  The specific references which have been amended are found on lines 518-530 and lines 535-542 |

**Editor Comments**

| A. Editor comment | I was not able to work out exactly where the “reduction in fertility” becoming a “medium reduction” came from. Is that the 3.0% in table S1 (which is 30 per women/ babies and therefore ‘medium’ risk by the diagram?) If so, why was ‘major puerperal infection’ (at 4.3 per 1000) not listed as a medium risk? |
| --- | --- |
| B. Response | The medium reduction in fertility did come from Table S1 (now Table 2 in the main text as per Editor comment 2). The issue is a formatting error in that the risk of ‘major puerperal infection’ was reported as an absolute risk difference of 4.3 per 1,000 whereas the reduction in live birth rate was reported as a percentage (3.0%), which equates to a difference of 30 per 1,000. These approximate small and medium risks, as described in Fig 1. |
| C. Changes made | The units in column 2 of Table 2 have been standardised, such that the reduction in live birth rate is reported as 30 per 1,000, rather than 3.0%. |
| D. Location of changes | Table 2. |

| A. Editor comment | When reading the major study cited for reduction in fertility after CS birth, it appears that a lot of the risk could be due to indications for the caesarean in the first place. Regardless, this discrete choice experiment is important in showing the value to the women of potential future fertility. |
| --- | --- |
| B. Response | Thank you for sharing your concerns. We have amended the manuscript to further address limitations in the data underlying a possible reduction in fertility after caesarean birth.  We had similar thoughts when designing the study and have now noted ongoing debate in the literature about the magnitude of the risk to fertility after caesarean birth in the Discussion (lines 357-363). |
| C. Changes made | The paragraph in the Discussion interpreting our findings in relation to future fertility now reads:  *“We also found that future fertility may be an important factor in low-risk pregnant women’s birth decisions. There is ongoing debate about the magnitude of the risk to fertility after caesarean birth. There is a need for further research on the possible risk to fertility after a caesarean birth given that existing data are from retrospective cohort studies, many of which poorly account for confounders including maternal age and comorbidities. [14, 16, 17, 460]. However, given our findings, obstetricians should consider informing their patients of the available evidence for this risk and its limitations.”* |
| D. Location of changes | Lines 357-363 |

| A. Editor comment | It would be ideal if S1 could be included in the main text. |
| --- | --- |
| B. Response | We agree that S1 contains information important to fully understand the methodology and have inserted its contents into the main manuscript. |
| C. Changes made | Table S1 is now Table 2 in the manuscript.  Figure S1 is now Figure 1 in the manuscript.  The remaining figures throughout the document and supplements have been renamed in according to the order mentioned in the main text, as per the PLOS ONE style guide. |
| D. Location of changes | Figure 1;  Table 2. |

| A. Editor comment | Minor – line 66 – should be “because it is unclear if IT would affect their decisions.” |
| --- | --- |
| B. Response | Amended, thank you! |
| C. Changes made | The line now reads:  *“Recovery is longer, and emerging evidence suggests that caesarean delivery reduces subsequent live birth rate, although this is not routinely discussed with patients because it is unclear if it would affect their decisions.”* |
| D. Location of changes | Line 68. |

| A. Editor comment | The exclusion criteria are reasonable, but it may be that the excluded cohorts differentially value risks after their high-risk pregnancy experiences. I agree with the reviewer in that this could be an avenue for further research. |
| --- | --- |
| B. Response | We agree that that the excluded cohorts may preference risks differently from our sample, and this is a limitation in the generalisability of our results. |
| C. Changes made | We have added the following statement into the Discussion to address this limitation of our study:  *“Furthermore, low-risk nulliparous women may weigh risks differently from high-risk pregnant women, or women with previous pregnancy experiences. This limits the generalisability of our findings and highlights an avenue for future research.”* |
| D. Location of changes | Lines 327-329. |

**Reviewer #1**

| A. Reviewer comment | Were there financial or other incentives offered for women who participated? |
| --- | --- |
| B. Response | No incentives were provided for women who participated. |
| C. Changes made | No changes made. |
| D. Location of changes | Not applicable. |

| A. Reviewer comment | Women preferred the risk profile of a caesarean section when considering unplanned intervention and maternal health; yet almost all women expected to have a vaginal delivery. Is long-term data available for these women? Is it known whether any of these women subsequently pursued a maternal request caesarean section or asked about this with their antenatal care providers? |
| --- | --- |
| B. Response | We agree that these are important questions, but unfortunately this data are not available as this question was beyond the scope of the study.  However, we asked participants whether they had discussed a caesarean birth with their doctor or midwife prior to completing the questionnaire, to which 18% of participants responded that they had. The major limitation of this datapoint is that women were able to complete the questionnaire from 28 weeks of gestation onwards, so some participants may have asked their care provider about a caesarean birth after completing the questionnaire.  We noticed that responses to this question were not included in the table of demographics – this was an error which has now been fixed. |
| C. Changes made | Addition of the row “Discussed caesarean birth with doctor or midwife before completing questionnaire – n (%)” to the table of demographic characteristics. |
| D. Location of changes | Table 3. |

| A. Reviewer comment | The disproportionate number of healthcare workers is appropriately addressed as a limitation. The authors note that this bias is mitigated as patients were not informed of the mode of delivery associated with each risk in the experiment. However, many healthcare workers (I would think almost all participants with midwifery or obstetric backgrounds) would know the mode of delivery associated with each risk from clinical experience. It is therefore perhaps worth rephrasing lines 292-295. |
| --- | --- |
| B. Response | We agree that this is an important limitation of our study and have rephased the lines accordingly. However, although healthcare workers are likely to have known the modes of delivery associated with each risk, the DCE was designed such that the risks of caesarean and vaginal birth were mixed between scenarios. Even if participants, for example, had a bias towards caesarean delivery, they could not select choices which overtly favoured this. |
| C. Changes made | The lines have been rephased, and now read:  *“One quarter of respondents also worked in the healthcare sector. Although the DCE was designed to mitigate against intrinsic biases favouring a caesarean or vaginal birth by not informing participants of the mode of delivery associated with each risk, healthcare workers may know these associations from their training or practice.”* |
| D. Location of changes | Lines 320-324 (formerly, lines 292-295). |

| A. Reviewer comment | Additionally, the health literacy of this cohort is likely to be much higher than the background population. It would be interesting to know whether the same risk preferences would be observed in a less educated background, and whether it would ultimately change birth preferences. This could be suggested as an avenue for future research. |
| --- | --- |
| B. Response | We agree with this limitation of our study, and that exploring the birth preferences of women from less highly educated backgrounds would be an important avenue for future research. |
| C. Changes made | The Discussion now includes the following statement:  *“The educational backgrounds of these participants may also have influenced their views, and further research should explore how risk preferences may differ in patients from different education backgrounds.”* |
| D. Location of changes | Lines 337-339. |

| A. Reviewer comment | Table 1: The text representing caesarean birth and vaginal birth under “Health of the baby” seem to be incorrect, ie. Medium risk of breathing difficulties and/or developing asthma or obesity in childhood should be listed as representing caesarean birth. |
| --- | --- |
| B. Response | Thank you. We apologise for this formatting error. |
| C. Changes made | The text representing caesarean and vaginal birth under “Health of the baby” have been corrected such that “Medium risk of breathing difficulties and/or developing asthma or obesity in childhood” is listed as representing caesarean birth and “Small risk of arm paralysis and/or brain injury” is listed as representing vaginal birth. |
| D. Location of changes | Table 1. |

| A. Reviewer comment | References are required for S1: “Major puerperal infection” and “Livebirth rate”. |
| --- | --- |
| B. Response | Thank you for alerting us to this referencing error, which occurred during our editing process. |
| C. Changes made | References have been provided for “Major puerperal infection” and “Livebirth rate” in Table 2 (formerly, Table S1).  The reference for “Major puerperal infection” is:  Liu S, Liston RM, Joseph KS, Heaman M, Sauve R, Kramer MS, et al. Maternal mortality and severe morbidity associated with low-risk planned cesarean delivery versus planned vaginal delivery at term. CMAJ. 2007;176(4):455-60. doi: 10.1503/cmaj.060870. PubMed PMID: 17296957; PubMed Central PMCID: PMC1800583.  The reference for “Livebirth rate” is:  Gurol-Urganci I, Cromwell DA, Mahmood TA, van der Meulen JH, Templeton A. A population-based cohort study of the effect of Caesarean section on subsequent fertility. Hum Reprod. 2014;29(6):1320-6. Epub 20140429. doi: 10.1093/humrep/deu057. PubMed PMID: 24781430. |
| D. Location of changes | Table 2. |

**Reviewer #2**

| A. Reviewer comment | Clarify how the randomisation of the choice sets was handled to ensure balanced presentation across participants. |
| --- | --- |
| B. Response | The order of choice sets was randomised for each participant using the Qualtrics® question order randomisation function, although each participant was shown the same eight choice sets which were developed to be balanced and orthogonal using the ‘AlgDesign’ package for R software. We have clarified this in the Methods. |
| C. Changes made | Under the subheading of Questionnaire design, a paragraph now reads:  *“Participants were asked to answer nine choice sets, the eight choice sets which comprised the fixed DCE design, and a repeat choice set (consistency test), included to test whether participants gave consistent responses. The order of the choice sets was randomised for each participant using the Qualtrics® question randomisation function.”* |
| D. Location of changes | Lines 190-194 |

| A. Reviewer comment | Provide more detail on how the free-text responses from the pilot phase were used to refine the questionnaire. |
| --- | --- |
| B. Response | The only change made to the questionnaire after the pilot interviews was insertion of the risk difference infographic to help participants weigh the risks, in response to feedback that 47% of participants had difficulty choosing between the risks. We have adjusted the Methods to clearly articulate this. We have now also addressed that no changes were made to the questionnaire in response to 9% of interview participants suggesting that the number of attributes in each scenario should be reduced, because difficulty choosing between the six attributes risks may reflect the complexity of the decision.  We accepted a risk that some participants may lose interest throughout the questionnaire, and randomised the order of choice sets (as described above) to mitigate any systematic biases that this may have caused. |
| C. Changes made | The section of the manuscript which addresses changes made to the manuscript after the pilot interviews now includes a paragraph which reads:  *“In the descriptive analysis of difficulties reported by pilot interview participants, one third (29.4%) reported no difficulties, and almost half (47.1%) described difficulty choosing between the risks. Only one quarter of participants provided any suggestions for improvement (Table S4), and the most common suggestion made by 9% of participants was to reduce the number of risks in each scenario. No changes were made to the choice sets following this feedback as difficulty choosing between the numerous included risks may reflect the inherent complexity of the birth decision. However, the questionnaire was amended to include the risk difference infographic (Figure 1) on every page to help participants weigh the risks.”* |
| D. Location of changes | Lines 238-246. |

| A. Reviewer comment | Discuss any potential biases or limitations in the sample selection and how they might impact the generalisability of the findings. |
| --- | --- |
| B. Response | This important feedback was also noted by the Editor. In response, we have included discussion that the educational and occupational backgrounds of the participants, and their low-risk pregnancy status, may limit the generalisability of our findings. We have also suggested that the risk preferences of patients who were not well-represented in our sample should be a focus of future research, including patients from lower educational backgrounds, multiparous women and patients with high-risk pregnancies. |
| C. Changes made | A paragraph in the limitations section of the manuscript now reads:  *“One quarter of respondents also worked in the healthcare sector. Although the DCE was designed to mitigate intrinsic biases favouring a caesarean or vaginal birth by not informing participants of the mode of delivery associated with each risk, healthcare workers may know these associations from their training or practice. The educational backgrounds of these participants may also have influenced their views, and further research should explore how risk preferences may differ in patients from different educational backgrounds.*  *Furthermore, low-risk nulliparous women may weigh risks differently from high-risk pregnant women, or women with previous pregnancy experiences. This limits the generalisability of our findings and highlights an avenue for future research.”* |
| D. Location of changes | Lines 320-329. |
| A. Reviewer comment | Reporting p-values helps identify significant differences in baseline characteristics that might impact the study’s outcomes. However, consider discussing these differences in the context of their potential impact on the results |
| B. Response | We agree that more discussion on how differences in the baseline characteristics between the interview and online survey samples is warranted, as the few differences found highlight an important limitation in our study: that pilot interview participants may had a better understanding of the risk described in our DCE, or otherwise weighed the risks differently. |
| C. Changes made | We have included additional discussion of the impact of the differences in baseline characteristics between the pilot interview and online survey samples:  *“As a more highly educated sample piloted the questionnaire, it is unclear how well the online survey participants understood the risks presented and this may limit validity of the online survey results.”* |
| D. Location of changes | Lines 334-336. |

| A. Reviewer comment | As an alternative to p-values, or to complement them, consider reporting standardised differences. This approach focuses on the magnitude of differences rather than just statistical significance. |
| --- | --- |
| B. Response | In response to this comment and the suggestion below, we have reported conditional odds ratios, 95% confidence intervals and p-values throughout the paper. However, we have elected to report p-values in isolation in our baseline characteristics table. By comparing the baseline characteristics of the interview and online survey groups, we intended to calculate the probability that demographics between the groups differed systematically because this is likely to affect interpretation of our results. This is achieved by a p-value alone, and implications of these findings on our results are discussed on lines 330-336. We agree that the magnitude of differences in demographics between the interview and survey groups is an important consideration when interpreting the results, but this can be determined by directly comparing the characteristics in Table 2. We believe that adding standardised differences would not be of additional value as we know the exact characteristics of the populations sampled. In addition, several journals and guidelines, such as STROBE, recommend against reporting of effect measures and p-values in descriptive tables of observational studies (such as “Table 1” of sample characteristics) because a) studies are often underpowered to identify statistically significant differences in baseline characteristics, b) differences in baseline characteristics that are not statistically significant may be clinically important, and c) most such differences can be inferred from the Table from the estimates of the groups.  We hope you can agree with our response. |
| C. Changes made | No changes made. |
| D. Location of changes | Not applicable. |

| A. Reviewer comment | Report the magnitude of the measure of association, 95% CI, and p-value together throughout the paper (including Table S5). |
| --- | --- |
| B. Response | We have adjusted Table S5 and the associated figures reported throughout the document accordingly.  Incidentally, while calculating these p-values, we found an error in the interview participant data set which affected one row of data and resulted in minor differences to the odds ratios for this sensitivity analysis. We have corrected this error and updated statistics throughout the document accordingly. The error did not affect statistical significance or change interpretation of any outcome for this sensitivity analysis. |
| C. Changes made | P-values have been added to S5 Table, and are now reported alongside confidence intervals wherever effect measure estimates are given.  The conditional odds ratios and associated confidence intervals for the sensitivity analysis for interview participants only have been updated in Table S5, and where mentioned throughout the document. |
| D. Location of changes | Addition of p-values to: S5 Table and everywhere outcomes are reported throughout the paper.  For sensitivity analysis of pilot interview participants, the following locations have been changed: S5 Table and line 283. |

| A. Reviewer comment | In Table S1, use the term “Relative Risk or Odds Ratio” for the third column. |
| --- | --- |
| B. Response | We agree that this is a more correct title for the column. |
| C. Changes made | The title of column 3 of Table 2 (formerly Table S1) has been changed to “Relative risk or odds ratio” |
| D. Location of changes | Table 2. |
